# Simulation reduces navigational errors in cerebral angiography training

**DOI:** 10.1186/s41077-020-00125-1

**Published:** 2020-06-12

**Authors:** Oleksiy Zaika, Mel Boulton, Roy Eagleson, Sandrine de Ribaupierre

**Affiliations:** 1 Anatomy & Cell Biology, Western University, London, Canada; 2 Schulich School of Medicine and Dentistry, Western University, London, Canada; 3 Clinical Neurological Sciences, Western University, London, Canada; 4 Electrical & Computer Engineering, Western University, London, Canada

## Abstract

**Background:**

Simulation-based medical education (SBME) is growing as a powerful aid in delivering proficient skills training in many specialties. Cerebral angiography (CA), a spatially and navigationally challenging endovascular procedure, can benefit from SBME by training targetable skills outside of the Angiosuite. In order to standardize and specify training requirements, navigational challenges and needs have to be identified. Furthermore, to enable successful adoption of these strategies, simulation adoption barriers, such as necessity of supervisory resources, must be reduced. In this study, we assessed the navigational challenges in simulated CA through a self-guided novice training program.

**Methods:**

Novice participants (*n* = 14) received virtual reality (ANGIO Mentor, Simbionix) diagnostic cerebral angiography training and were tested on a right middle cerebral artery aneurysm case over 8 sessions with a reference instructional outline. The navigational trajectories for the guidewire and catheter were analyzed and rates in erroneous vessel access were analyzed. Participants were given a Mental Rotations Test (MRT) and were analyzed based on MRT performance.

**Results:**

After 8 sessions, there was a significant (*p* < 0.05) reduction on navigational error prevalence. The L-SUB and L-CCA saw the biggest drop in erroneous access, whereas the R-ECA, the biggest consumer of error time, saw no changes in access frequency. Individuals with high MRT score performed much better (*p* < 0.05) than those with low MRT score.

**Conclusions:**

Through self-guided simulation training, we demonstrated the navigational challenges encountered in simulated CA. To establish better assessments and standards in medical training, we can create self-guided training curricula aimed at correcting errors, enabling repetitive practice, and reducing human resource needs.

## Background

Integration of simulation-based medical education (SBME) into traditional training approaches has the potential to drastically improve the rate of clinical skill acquisition and reduce overall strain on the medical system. One of the arguable strengths of SBME lies in creating a safe environment for trainees to make and learn from mistakes that would otherwise have been harmful for patients [1]. This is especially significant in skillsets consisting of steep learning curves, such as those found in cerebral angiography (CA) training.

Development of endovascular proficiency in CA requires multimodal acuity due to the limited visuospatial feedback—a distal guidewire tip is hard to navigate through the lumen of 3-dimensional vascular anatomy using temporally constrained 2-dimensional fluoroscopic imaging. These motor and visuospatial skills comprise the core of CA and should ideally be trained extensively outside of the Angiosuite (hybrid operating room). Unfortunately, current angiography fellowship programs still rely on traditional Angiosuite-based training methods for skill development despite the availability of haptic simulators.

One of the most established high-fidelity simulators available is the ANGIO Mentor from Simbionix. This task-trainer has been shown to have construct validity [2], improve psychomotor learning [3], reduce procedure and fluoroscopy time [3–5], and enhance resident performance [6]. Although impressive, this has not led to a widespread adaptation of simulation in angiography training. Introduction of SBME is generally limited by scarcity of human resources, logistical barriers, and laborious coordination [7], however, its particularly lagging implementation in CA may be attributed to insufficient identification and standardization of targetable skills.

**Fig. 1:**
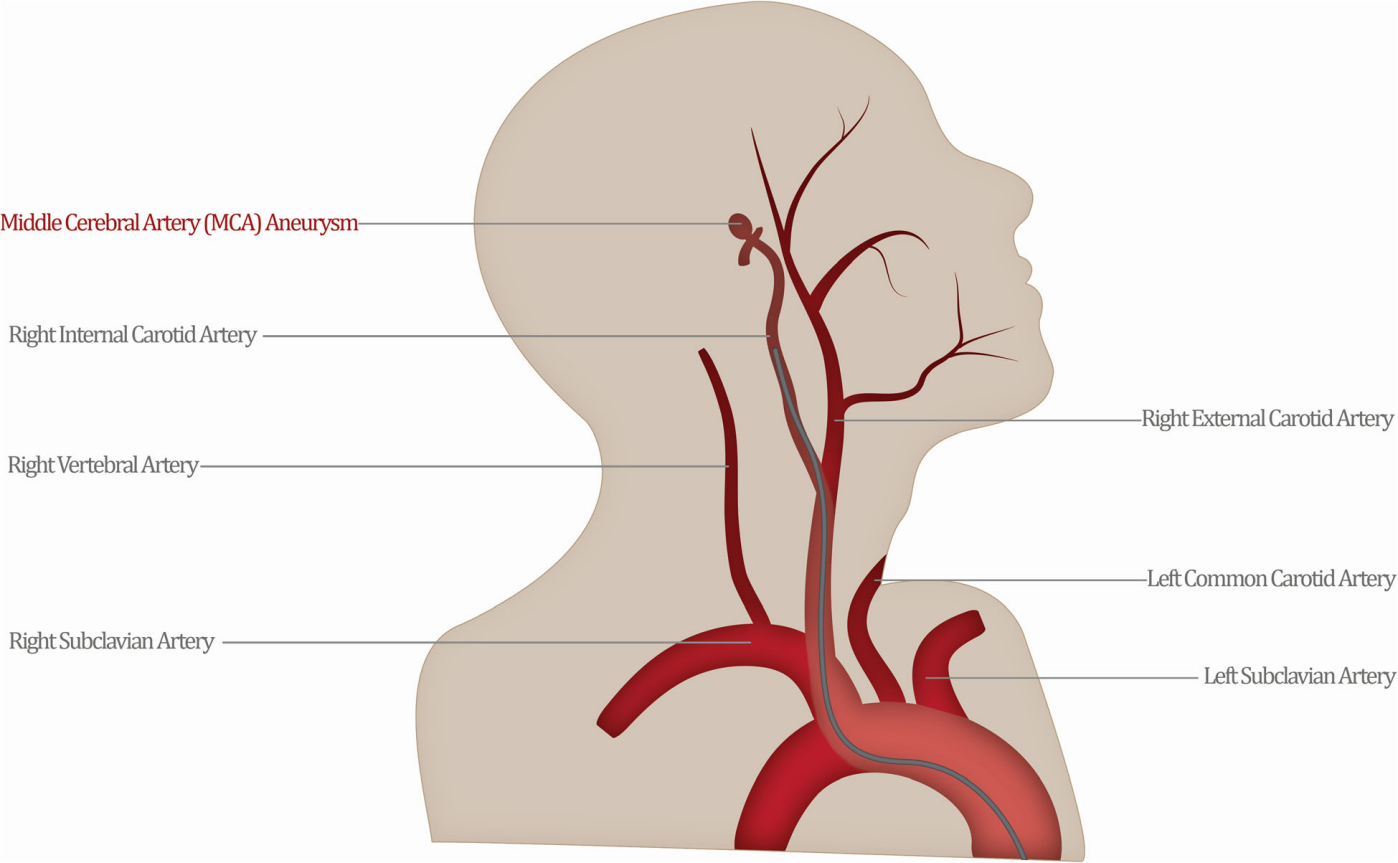
An expert interventionalist would establish the most direct path to the site of the aneurysm (highlighted), avoiding any deviations and bifurcations that would lead to lost procedural efficiency (red)

Recognizing training obstacles for novice trainees is a key component in tailoring their learning experience and providing standardized goals. Some of the most basic errors committed by novices are purely navigational—limited imaging, inexperience, and excessive tool manipulation [8, 9] can cause major errors [10] and deviations from their expected trajectory (Fig. 1). However, most proficiency assessments still focus on subjective grading schemas and program-based case exposure requirements.

The literature does not currently explain how junior trainees develop navigational competence, despite its necessity for expert performance. This level of granularity in skill development has been difficult to measure in the Angiosuite due to the multifaceted operational components of the procedure, such as fluoroscopy time and contrast use. Spatial ability may play an important role as it has been shown to be correlated with greater performance across a variety of specialties including laparoscopy [11], colonoscopy [12], and sonography [13] but is not used in determining training needs.

Creating opportunities for trainees to rehearse and make navigational errors in simulation, even without expert guidance, may lead to an overall reduction in their prevalence. We aim to understand the prevalent navigational errors in simulated CA and their potential for correction through an externally directed, self-guided simulation training program. Directed self-guidance allows the novice to manage their education independently under externally shaped content and context [14]. Furthermore, by established guidelines for this metric, it may be possible to decrease simulation adoption barriers by reducing physician mentoring stress and encouraging independent practice.

## Methods

### Participants

A total of 8 clinical anatomy graduate students and 6 residents in neurosurgery and radiology specialties were recruited for this study. The cohort was selected due to their unique combination of vascular anatomy competency and lack of technical endovascular training. Although this combination provided a uniform baseline of experience, it limited the number of participants available to be recruited. Participants were provided with a neurovascular overview, followed by a vascular anatomy labeling quiz. A grade cut-off of 80% was used to remain in the study. All participants met the required anatomy quiz score, resulting in no exclusions in the participants’ cohort.

### Materials

**Fig. 2:**
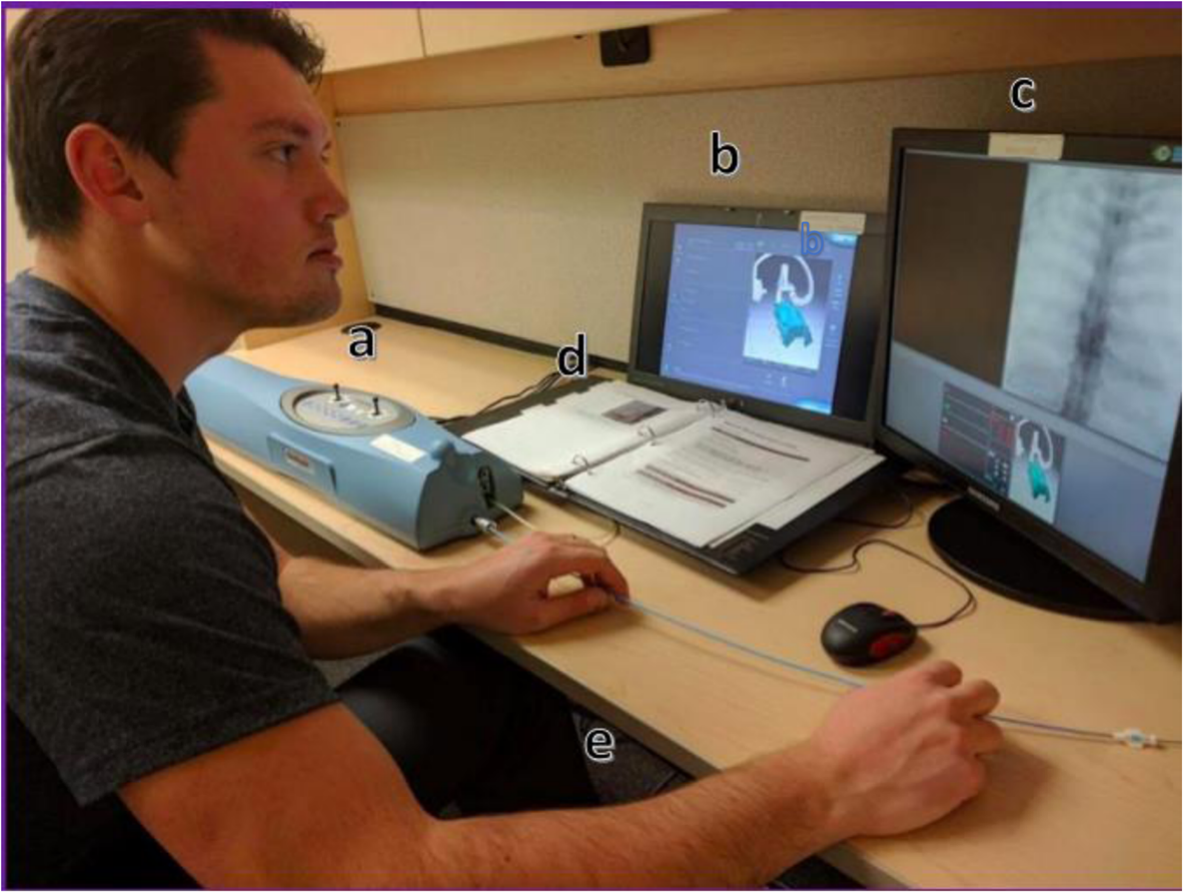
The ANGIO Mentor by Simbionix includes a simulation control panel (**a**), main tool interface (**b**), fluoroscopy screen (**c**), instructional booklet (**d**), and pedals (**e**)

A haptic feedback simulator, ANGIO Mentor by Simbionix (Fig. 2), was used to train, test, and collect data from the participants. Trainees were instructed on basic functionality of the control panel, including rotating fluoroscopy C-arm, shifting patient table, creating and clearing roadmaps, injecting contrast, activating fluoroscopy, and performing a digitally subtracted angiogram of the aneurysm. Two monitors were used to display the tool and patient state (b) and patient fluoroscopy imaging (c).

### Measures

#### Error criteria

The correct pathway for navigating to a right middle cerebral artery (R-MCA) aneurysm included the most direct endovascular trajectory, a route that is used clinically for accessing aneurysms on the right side of the brain—the arch of the aorta, brachiocephalic (i.e., R-SUB), right common carotid (R-CCA), and internal carotid arteries (R-ICA). A navigational error was deemed to be any deviation from this pathway into a neighboring artery. Thus, accessing the following areas with either the guidewire or the catheter was included as a navigational error—left subclavian (L-SUB), left common carotid (L-CCA), right vertebral (R-VERT), or right external carotid (R-ECA) arteries.

#### Chance design

The difficulty of entering one of the great vessels was assessed through chance. The guidewire was inserted blindly into the simulated patient and advanced the distance of the arch of the aorta. The resulting location of the guidewire was recorded. The tool was advanced for 50 cycles without guidewire rotation and for 50 cycles with a clockwise rotation per inch of insertion.

### Procedure

Participants were given two consecutive Vandenberg and Kuse [15] Mental Rotation tests (MRT) to assess visual-spatial skills. Each test contained 12 questions and was timed at 3 min. The scores were used to split participants post hoc into a low MRT group (score less than median) and high MRT group (score higher than median). There is evidence to suggest that comparing lower and upper quartiles may be more appropriate to contrast the scores [16], however, this was not possible in our sample size. MRT groups were compared to each other with respect to time spent in incorrect vessels.

Participants individually attended 8 weekly sessions, each of which consisted of an untimed practice case (left internal carotid artery (L-ICA) aneurysm) to familiarize participants with the equipment, and a timed test case (R-MCA aneurysm) with a step-wise instructional sheet. Session quantity and frequency were set to ensure a significantly spaced time frame for learning to occur on repeating cases. Participants could ask questions during practice; however, no assistance was provided during the test case. A case was deemed to be finished when the participant declared that they have achieved the last step in their instructions.

In assessing the capacity of navigational skills, several quantitative performance attributes were extracted from the simulation software. Using the participant performance log files, the locations of the tools throughout the procedures were used to calculate frequency and length of vessel access. Navigational errors, or access of incorrect vessels along the optimal vascular trajectory, were assessed using these markers. Time spent exploring incorrect vessels is likely to increase total fluoroscopy time and negatively impact patient outcomes [17].

### Data analysis

All data was automatically collected by the simulator and analyzed manually using Microsoft Excel. Vessel access timestamps were analyzed for frequency and compiled together to calculate duration of stay within each vessel.

The simulator data was exported and analyzed using Excel and SPSS 19. Timing and frequency of vessel access across 8 sessions were analyzed using a repeated-measures independent analyses of variance (ANOVA) with a Bonferroni correction. MRT influence was also assessed using a two-way repeated-measures ANOVA. A statistical significance of *p* < 0.05 was used for all assessments.

## Results

### Time spent in incorrect vessels

**Fig. 3:**
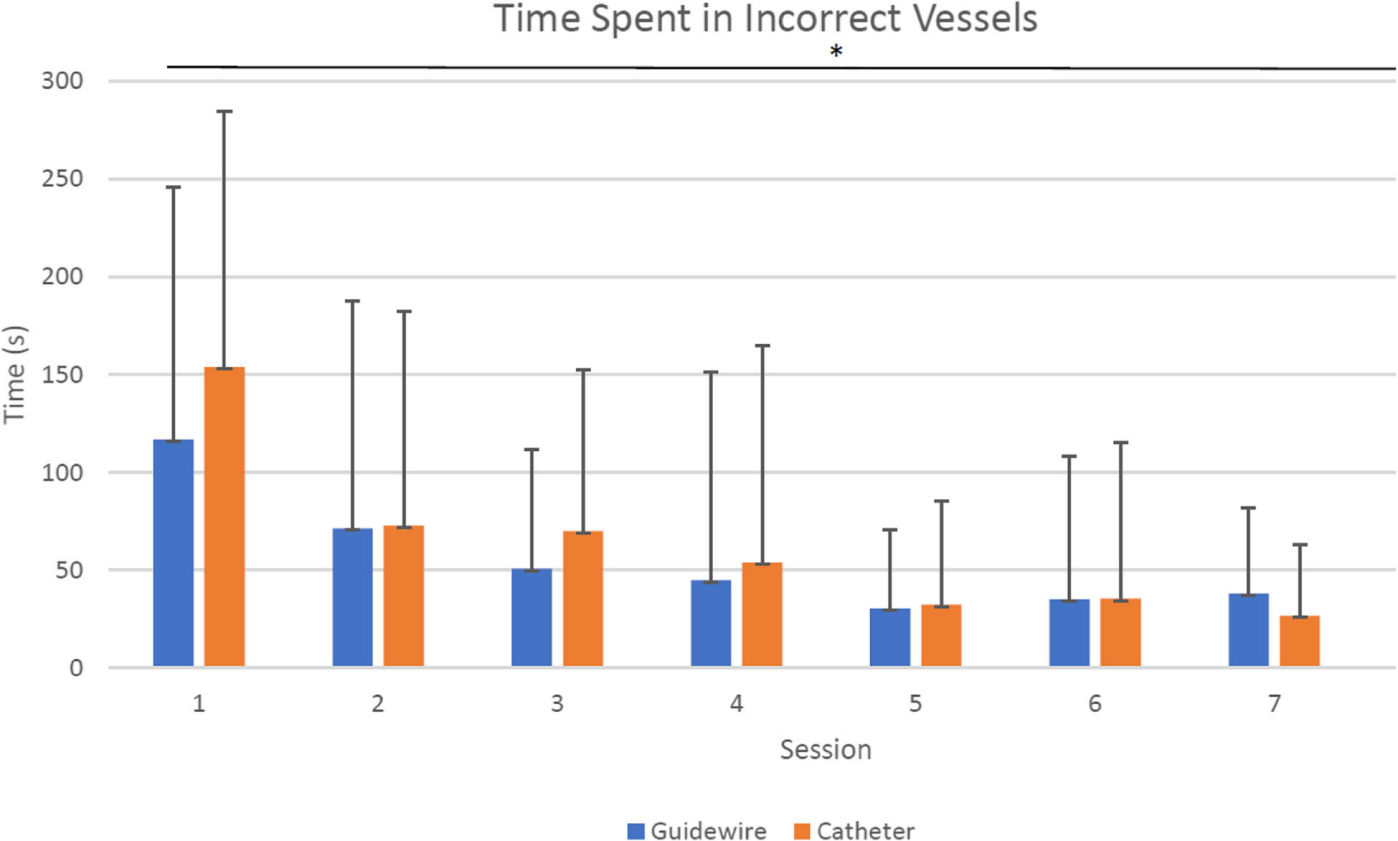
All participants had significantly lowered time wasted in incorrect vessels with both catheter and guidewire over 8 sessions

All participants had significantly improved performance over 8 sessions by reducing their time spent in incorrect vessels (*p* < 0.05) while navigating through a diagnostic R-MCA cerebral angiography case with both guidewire and catheter (Fig. 3). The average time spent in incorrect vessels with the guidewire dropped from 153 to 44 s using the catheter and from 117 to 44 s using the guidewire (Fig. 3). Comparing this result with our previous study assessing total procedural times [5], it is indicative that one of the main reasons users improve their procedural time is their lowered time wasted on navigational mistakes.

### Vessel access time

**Fig. 4:**
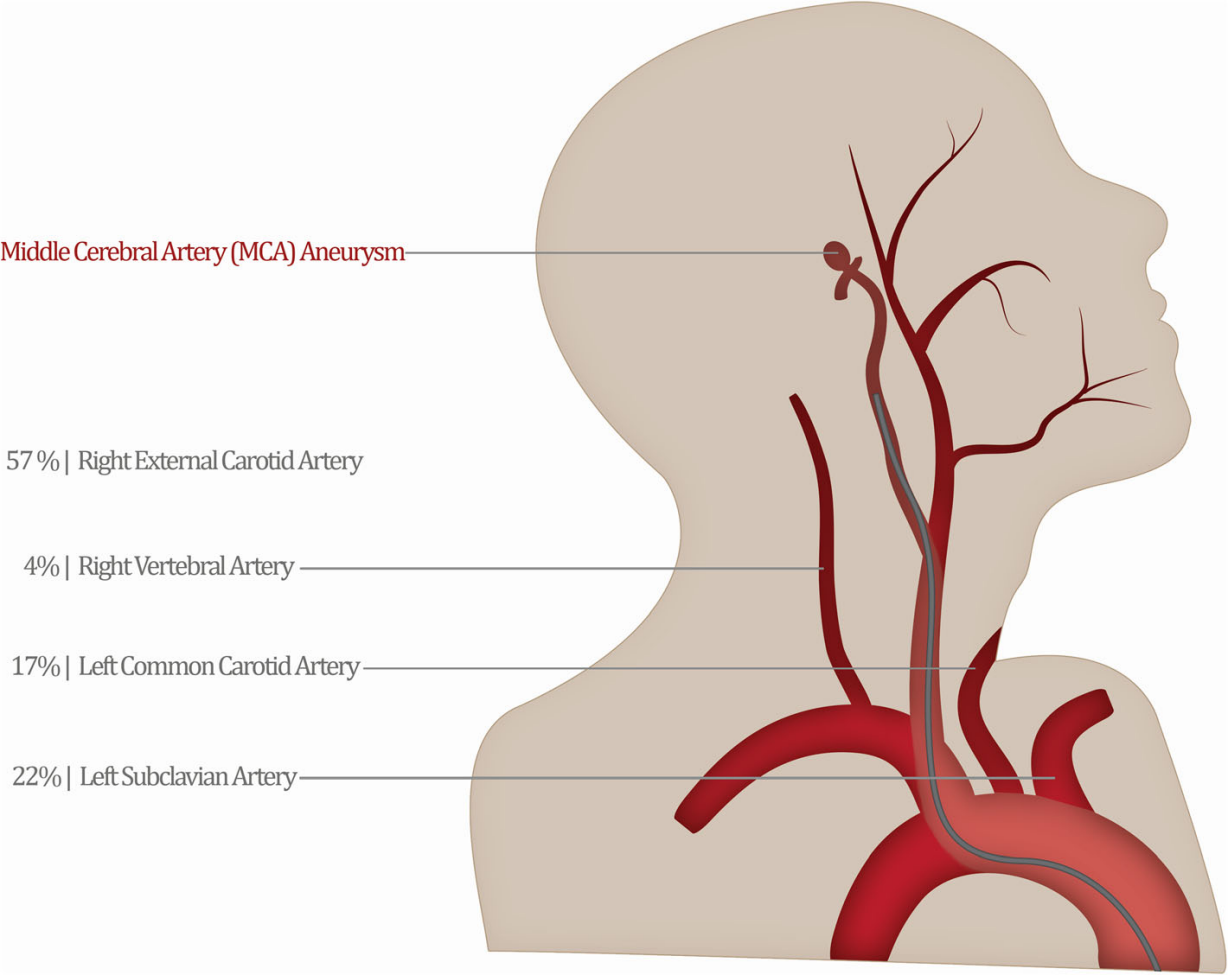
Time spent in each incorrect vessel differed drastically from its access frequency, with the R-ECA requiring the most time of all mistakes

When analyzed by vessel, time spent in each vessel differed greatly. Based on the participants’ first-session performance, accidentally entering the R-ECA resulted in the highest amount of time wasted, compared to lower duration in L-CCA, L-SUB, and R-VERT. The participants spent a total of 362 s (22%) in the L-SUB artery, 281 s (17%) in the L-CCA artery, 65 s (4%) in the R-VERT artery, and 931 s (57%) in the R-ECA artery (Fig. 4). By session 8, participants were spending a total of 35 s (6%) in the L-SUB artery, 15 s (3%) in the L-CCA, no time in the R-VERT artery, and 509 s (91%) in the R-ECA. There was an observed reduction in all erroneous vessel access; however, the ratio of errors increased in the R-ECA, indicating that this navigational and spatial issue is not as easily resolved with general practice. The concern is twofold in that this particular mistake could cause the most damage due to the small lumen of the R-ECA. The frequency of navigational errors may not be the dominant concern, since how those errors are corrected seems to have a larger effect on performance: a novice that spends more time readjusting within the R-ECA may be in need of more training than their frequency-matching colleague.

### Frequency of incorrect vessel access

**Fig. 5:**
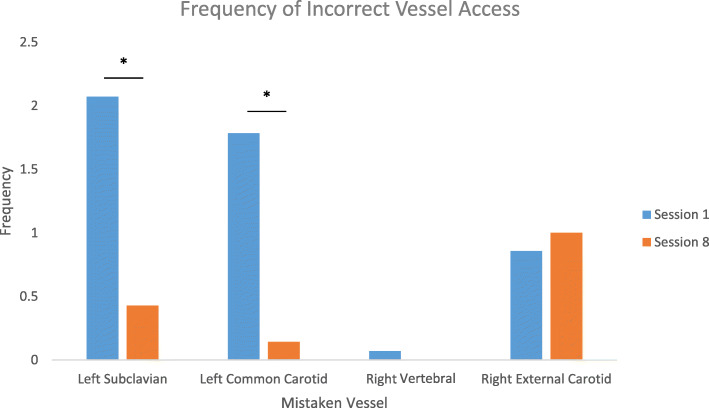
Frequency of accessing the L-SUB and L-CCA dropped significantly (*p* < 0.05) from session 1 to session 8; however, this change was not observed in the R-ECA access frequency

The frequency of incorrect vessel access varied tremendously based on the anatomical area. The alternate pathways off the arch of the aorta saw the highest frequency of erroneous access, with the left subclavian artery and left common carotid arteries being entered with guidewire on average 2.1 and 1.8 times in the first session. The R-VERT access time was insignificant (0.1 times) and its position relative to the trajectory of the guidewire would not be considered a navigational obstacle. The access to the R-ECA, although accessed only 0.9 times on average in the first session, presents a large issue for training professionals (Fig. 5).

By the last session, the participants were on average accessing most erroneous vessels with a lower frequency: the L-SUB 0.5 times, the L-CCA 0.6 times, R-VERT 0.2 times, and R-ECA 0.2 times. The L-SUB, L-CCA, and R-ECA had a statistically lower access rate (*p* < 0.05), whereas R-VERT did not have a significant change.

The drop in access frequency for some of the incorrect vessels, such as L-CCA and L-SUB, points to the concept that trainees are developing a better understanding of the spatial trajectory and can make corrections throughout training. Unchanged levels in right vertebral artery access confirm this is not a major obstacle in accessing the R-ICA. The major issue seemed to arise from accessing the R-ECA while attempting to traverse into the R-ICA. Over the 8 sessions, there was no significant decrease in R-ECA access frequency. This result identifies that either (a) this error cannot be fixed with just 8 training sessions or (b) this error needs to be addressed in a targeted manner. The anatomy of the anterior-posterior overlap between the external and internal carotids suggests that trainees may need further training to better formulate the spatial relationship in their fluoroscopy interpretation.

### Chance of access

Upon insertion, the guidewire and catheter were consistently simulated facing in the medial direction. A blind insertion of guidewire into the system over 50 cycles showed no accidental access into the L-CCA or L-SUB, with all trajectories ending up at the base of the aorta. Once a steady rotation was applied over 50 cycles, the guidewire accessed the left subclavian artery 21 times (42%) and only finished at the base of the aorta 29 times (48%). This data could explain why even high spatial performers make the mistake of accessing the wrong great vessels at the start of the procedure. As they advance their tools and rotate them, their chance of accessing the wrong vessel increases considerably. Novices tend to use too many movements while performing the procedure [18] and may benefit from simulated practice on smoother rotations and motions using endovascular tools.

### Spatial ability groups

**Fig. 6:**
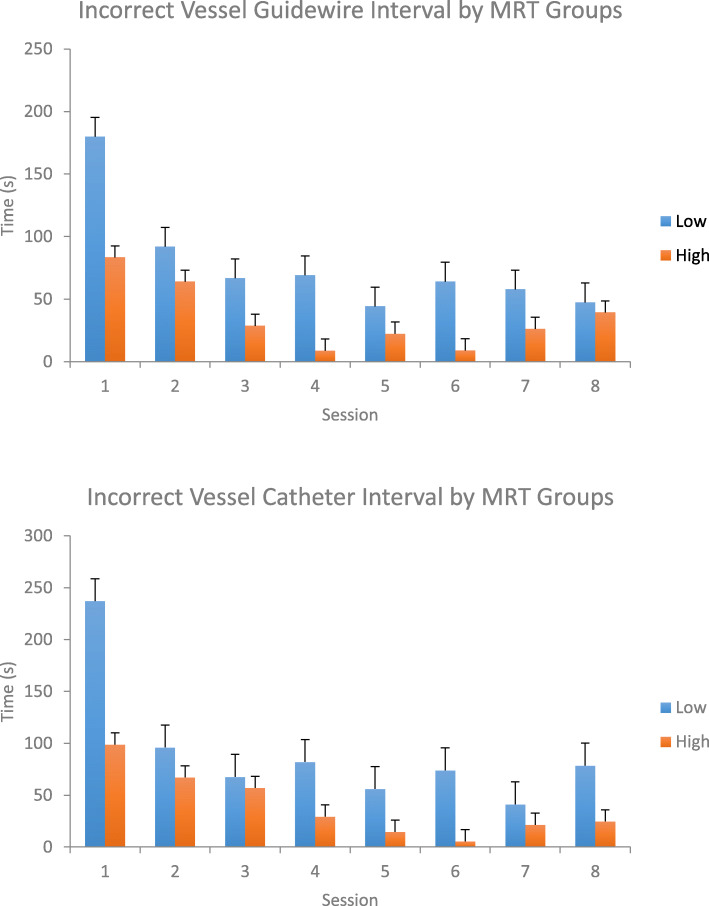
Participants with a low MRT score spent significantly more time exploring incorrect vessels with the guidewire and catheter, compared to the high MRT participants

There were 7 participants per group, with the low MRT group (MRT ≤ 13) averaging 9.21 and the high MRT group (MRT ≥ 14) averaging 15.86. Individuals with low MRT score spent significantly more time (*p* < 0.05) in the incorrect vessels than those with high MRT score, shown with both the catheter and guidewire timing (Fig. 6). In our analysis of MRT groups, the results were quite consistent with previous research [5].

## Discussion

In the field of cerebral angiography, simulation training has already been shown to drastically reduce fluoroscopy and procedural time in novice trainees [3–5]. Although useful in assessing overall performance, these metrics do not provide insight into how and why the trainees improve, dismissing the incredible capacity to target specific skills using SBME. The data generated by this study identifies simulation’s potential in being used to gain vascular familiarity, navigate endovascular tools, and interpret fluoroscopic images. Just as importantly, although interventional fellow training in simulation offers no direct benefit to the patient (compared to training in the Angiosuite), training basic skills independently may significantly reduce physician mentoring stress and reallocate expert interventionalist time back to the patient. By alleviating some of these implementation barriers, complementary simulation training can make skill acquisition much more affordable, accessible, manageable, and measurable. Novice fellows may see the highest benefit if they are able to complete their interventional training at a higher level of competency as a result of complementary practice.

Although the improvement in navigational skills in simulation is encouraging, it should be recognized that this study was focused on simulation-only training and testing. The translation of skills learned in simulation into the Angiosuite would be the true test of its potential and would be necessary to create an adoptable training curriculum. In its development, the training module would greatly benefit from increased focus on the problematic vessels, such as the external carotid artery, in order to standardize performance across the entire endovascular pathway. Additional resources and continued education can be provided to fellows who find the procedure challenging (i.e., low MRT individuals).

As specialties continue to turn to personalized and supplementary training curricula, a paradigm already seeking momentum in medicine [19], there will be an increased need for unsupervised, self-guided simulation practice. The evolution of medical education towards a competency-based learning model will require standardized performance markers established through a strong collaboration between researchers, expert interventionalists, and medical educators. This approach to training would enable data-driven analyses of performance to reveal new markers of performance essential for improved patient safety. Furthermore, medical educators can create personalized approaches to target common errors committed by interventional fellows as well as challenging vascular variations. As this research progresses, navigational schemas for vessel difficulty would create a strong personalized curriculum adaptable to the deliberate training needs of the fellow and the outcome needs of the patient.

## Conclusion

Simulation-based angiography training is the key to establishing standardized training requirements to target core skillsets in junior fellows. Trainees should be able to learn the essential navigational and motor skills independently using simulation and transfer those skills to a clinical setting under expert guidance. By identifying areas of navigational difficulty, the limited training resources can be better allocated towards focused needs of junior interventionalists.

## Data Availability

Raw data from the simulator is not publicly available in line with ethical approval related to potentially identifiable information.

## Abbreviations

SBME: Simulation-based medical education
CA: Cerebral angiography
L-SUB: Left subclavian artery
L-CCA: Left common carotid artery
R-SUB: Right subclavian artery
R-VERT: Right vertebral artery
R-CCA: Right common carotid artery
R-ICA: Right internal carotid artery
R-ECA: Right external carotid artery
R-MCA: Right middle cerebral artery
